# Network-based approach to identify prognosis-related genes in tamoxifen-treated patients with estrogen receptor-positive breast cancer

**DOI:** 10.1042/BSR20203020

**Published:** 2021-09-03

**Authors:** Yanyan Wang, Xiaonan Gong, Yujie Zhang

**Affiliations:** 1 Department of Breast Surgery, The Second Affiliated Hospital, Zhejiang University School of Medicine, 88 Jiefang Road, Hangzhou 310009, China; 2 Department of Orthopedic Surgery, The Second Affiliated Hospital, Zhejiang University School of Medicine, 88 Jiefang Road, Hangzhou 310009, China

**Keywords:** biomarker, estrogen receptor-positive breast cancer, tamoxifen, weighted gene co-expression network analysis

## Abstract

Tamoxifen is an estrogen receptor (ER) antagonist that is most commonly used for the treatment of ER-positive breast cancer. However, tamoxifen resistance remains a major cause of cancer recurrence and progression. Here, we aimed to identify hub genes implicated in the progression and prognosis of ER-positive breast cancer following tamoxifen treatment. Microarray data (GSE9893) for 155 tamoxifen-treated primary ER-positive breast cancer samples were obtained from the Gene Expression Omnibus database. In total, 1706 differentially expressed genes (DEGs), including 859 up-regulated and 847 down-regulated genes, were identified between relapse and relapse-free samples. Weighted correlation network analysis clustered genes into 13 modules, among which the tan and blue modules were the most significantly related to prognosis. From these two modules, we further identified and validated two prognosis-related hub genes (G-rich RNA sequence binding factor 1 (*GRSF1*) and microtubule-associated protein τ (*MAPT*)) via survival analysis based on several publicly available datasets. High expression of *GRSF1* predicted poor prognosis, whereas *MAPT* indicated favorable outcomes in ER-positive breast cancer. Using breast cancer cell lines and tissue samples, we confirmed that *GRSF1* was significantly up-regulated and *MAPT* was down-regulated in the tamoxifen-resistant group compared with the tamoxifen-sensitive group. The prognostic value of *GRSF1* and *MAPT* was also verified in 48 tamoxifen-treated ER-positive breast cancer patients in our hospital. Gene set enrichment analysis (GSEA) suggested that *GRSF1* was potentially involved in RNA degradation and cell cycle pathways, while *MAPT* was strongly linked to immune-related signaling pathways. Taken together, our findings established novel prognostic biomarkers to predict tamoxifen sensitivity, which may facilitate individualized management of breast cancer.

## Introduction

Breast cancer is a heterogeneous cancer, displaying a variety of molecular features, prognostic patterns, and therapeutic responses [1]. Up to two-thirds of all cases express estrogen receptor (ER), and can be treated using hormone-based therapy. Tamoxifen is a first-generation selective ER modulator that competes with estradiol to bind to ERs, thereby antagonizing the effects of estrogen and inhibiting the growth and proliferation of tumor cells [2]. The administration of tamoxifen greatly minimizes the risk of recurrence of ER-positive breast cancer, particularly for premenopausal women [3]. Unfortunately, approximately 40% of ER-positive patients are less sensitive to tamoxifen treatment, and will eventually relapse with endocrine-resistant phenotypes [4,5]. To date, the exact mechanisms of tamoxifen insensitivity in breast cancer remain largely unknown, and tamoxifen-resistant cancer is difficult to treat, due to lack of therapeutic targets. Since tamoxifen therapy fails for a large number of patients, there is an urgent need to elucidate the molecular mechanisms of tamoxifen resistance, particularly to identify novel potential genes for monitoring treatment efficacy and predicting prognosis.

Co-expression analysis has recently emerged as a powerful technique for mining gene expression profiles in various cancers. As an effective bioinformatics approach, weighted gene co-expression network analysis (WGCNA) is increasingly applied to explore synergistically altered gene sets, and to identify candidate biomarkers associated with clinical parameters [6–8]. In breast cancer, several studies have utilized WGCNA to identify hub genes closely related to clinicopathological traits (e.g., tumor size, grade, and molecular subtypes) and survival outcomes. For example, Tang et al*.* found that elevated expression of ASPM, TTK, and CDC20 conferred a poorer prognosis in breast cancer [9], and Jiang et al. identified six key genes (*CA12*, *MLPH*, *FOXA1*, *GATA3*, *XBP1*, and *MAGED2*) that could serve as biomarkers for the prediction of better chemotherapeutic responses and favorable prognosis in patients with breast cancer [10].

Accordingly, in the present study, we conducted an integrated analysis based on WGCNA to screen out novel prognostic biomarkers associated with tamoxifen response in breast cancer patients. In addition, the expression levels and the prognostic value of candidate hub genes were determined *in vitro* using cell lines and clinical tissue samples. Our findings may shed light on the underlying mechanisms of tamoxifen resistance in breast cancer, and may provide new prognostic markers to accurately predict tamoxifen response.

## Materials and methods

### Data collection and processing

The gene expression profile GSE9893 was obtained from the Gene Expression Omnibus database (https://www.ncbi.nlm.nih.gov/geo/), and evaluated using the GPL5049 platform [11]. The dataset GSE9893 comprised 155 tamoxifen-treated primary breast cancer samples, of which 52 cases developed recurrent disease (designated the tamoxifen-resistant group). Robust multiarray average background correction and log_2_ conversion were performed using the ‘affy’ R package. Probes were mapped on to genes using Affymetrix annotation files. Genes matching with multiple probes were averaged to obtain the expression level of the gene. Probes corresponding to multiple genes were deleted.

### Analysis of differentially expressed genes

The ‘limma’ R package with the Empirical Bayes method was employed to identify differentially expressed genes (DEGs) between relapse and relapse-free samples. Statistically significant DEGs were defined as |log_2_ FC| > 1 and *P*<0.01. The results were visualized by plotting a volcano plot using the ‘ggplot2’ package in R.

### Functional enrichment analysis

After identifying DEGs related to tamoxifen sensitivity in breast cancer, the STRING database (https://string-db.org) was used to perform Gene Ontology (GO) and Kyoto Encyclopedia of Genes and Genomes (KEGG) pathway enrichment analysis to determine the biological functions and pathways of tamoxifen resistance-related genes. The cutoff was set at an adjusted *P-*value of less than 0.05.

### Co-expression network construction by WGCNA

Co-expression networks were established to explore modules involved in tamoxifen sensitivity in breast cancer using the ‘WGCNA’ package in R. First, outlier samples were detected using the sample network method. The soft threshold for WGCNA construction was selected such that the constructed network mainly included genes with strong correlations. We then transformed adjacency to a topological overlap matrix (TOM) to examine the connectivity of the network, followed by hierarchical clustering construction based on the TOM dissimilarity, to categorize genes with similar expression profiles into modules. The minimum module size for the gene dendrogram was 50, and other parameters were set to the default values. Finally, analyses of module eigengene, gene significance, and module–trait relationships were performed to identify clinically significant modules.

### Selection of hub genes

Hub genes were identified as highly interconnected genes in a module of WGCNA. Tan and blue modules were considered key modules because they were closely related to the metastasis and recurrence of tamoxifen-resistant breast cancer. Hub genes were then screened out according to the absolute value of the Pearson’s correlation co-efficient. The modules of significance were visualized using Cytoscape (version 3.6.0; https://cytoscape.org/). The Cytoscape plugin ‘molecular complex detection’ (MCODE) was applied to detect the most important subnetworks, with a degree cutoff = 2, node score cutoff = 0.2, k-core = 2, and max depth = 100 set as the criteria [12].

### Validation of prognosis-related hub genes by survival analysis

Survival analysis was performed with hub genes to further identify prognosis-associated genes using The Cancer Genome Atlas (TCGA) breast cancer dataset. All breast cancer patients were classified into two groups according to the expression level of a particular gene (high versus low). Kaplan–Meier survival analysis was then performed to compare the overall survival between these groups using the ‘Survival’ package in R. We further validated the survival results associated with each of the candidate hub genes in three independent ER-positive breast cancer cohorts, comprising the datasets GSE3494 and GSE25066, containing 201 and 296 ER-positive breast cancer patients, respectively, as well as the GSE9893 dataset. Results with *P-*values <0.05 were considered statistically significant.

### Cell culture

The breast cancer cell line MCF-7 was purchased from the American Type Culture Collection (Manassas, VA, U.S.A.) and routinely maintained in Dulbecco’s modified Eagle’s medium (DMEM) with 10% fetal bovine serum. Tamoxifen-resistant cells (MCF-7/TAM) were established by continuously culturing MCF-7 cells in the presence of 4 μM 4-hydroxy-tamoxifen (Sigma–Aldrich, Missouri, U.S.A.) for 6 months. All cells were grown at 37°C in an atmosphere containing 5% CO_2_.

### Reverse transcription quantitative polymerase chain reaction

RNA extraction was performed using TRIzol reagent (TaKaRa, Otsu, Japan). Total RNA was reverse transcribed to cDNA using a PrimeScript RT reagent kit (TaKaRa). Subsequently, qPCR was performed with harvested cDNA using the SYBR Green PCR kit (TaKaRa). The relative mRNA levels were calculated using the 2^−ΔΔ*C*_t_^ method taking GAPDH as the internal control. The primers used for reverse transcription quantitative polymerase chain reaction (RT-qPCR) were as follows: G-rich RNA sequence binding factor 1 (*GRSF1*) forward, 5′-ACAGGGAAGAAATTGGTAATCG-3′ and reverse, 5′-ACCATCGTCTACTGCCCTTTC-3′; and microtubule-associated protein τ (*MAPT*) forward, 5′-AAAGACGGGACTGGAAGCG-3′ and reverse, 5′-GAATCCTGGTGGCGTTGG-3′.

### Preparation of ER-positive human breast cancer samples

Twenty-three tamoxifen-resistant and 25 tamoxifen-sensitive paraffin-embedded tumor samples were obtained from ER-positive breast cancer patients who underwent surgery at the Department of Breast Surgery, The Second Affiliated Hospital, Zhejiang University School of Medicine, during June 2012 to September 2019. Informed consent was obtained from all patients. The study was performed with the approval of The Human Research Ethics Committee of The Second Affiliated Hospital, Zhejiang University School of Medicine. The present study conformed to the Declaration of Helsinki.

### Immunohistochemistry

Paraffin-embedded tissue sections were dewaxed with xylene and rehydrated with ethanol, followed by antigen retrieval with EDTA (pH 9.0). Endogenous peroxidase was removed by adding 3% H_2_O_2_. The slides were incubated with goat serum and anti-GRSF1 (Abcam, MA, U.S.A., dilution 1:100) or MAPT (Abcam, dilution 1:400) primary antibodies overnight at 4°C. Detection was performed by incubating with horseradish peroxidase (HRP)-linked anti-Rabbit IgG and 3,3′-diaminobenzidine (DAB). The staining intensity was scored as follows: 0, negative; 1, weak; 2, moderate; 3, strong. The percentage of stained cells was scored into four grades: 0, < 5%; 1, 5–25%; 2, 25–50%; 3, >51%. Intensity and percentage scores were multiplied to obtain the final scores (0, 1, 2, 3, 4, 6, or 9), with a cutoff <3 versus ≥3 to indicate low versus high expression, respectively. Staining was scored by two independent observers in our hospital.

### Gene set enrichment analysis of hub genes

To explore the molecular mechanisms of identified hub genes on breast cancer, gene set enrichment analysis (GSEA) was carried out with the ER-positive TCGA dataset [13]. The samples were separated into low and high groups in accordance with degree of hub gene expression, and c2.cp.kegg.v5.2.symbols.gmt was selected as a reference gene set. A false discovery rate < 0.05 was designated as the cut-off criteria.

### Tumor immune estimation resource database analysis

Because of the essential role of immune infiltration in cancer initiation and progression, we used the tumor immune estimation resource (TIMER) online database to determine the association between tumor-infiltrating immune cells and each hub gene [14]. The six types of immune cells inferred by TIMER included B cells, CD4 T cells, CD8 T cells, dendritic cells, macrophages, and neutrophils. The levels of hub gene expression were visualized by log_2_ RSEM.

### Statistical analysis

All *in vitro* experiments were independently repeated three times. Two-tailed Student’s *t* tests were used to detect differences between groups using SPSS 17.0 software (IBM, NY, U.S.A.). In survival analysis, we used Kaplan–Meier analysis and a Cox proportional hazards regression model to ascertain whether candidate hub genes had an effect on prognosis. The hazard ratio (HR) and 95% confidence interval (CI) were calculated from the regression coefficients and survival curves were plotted using GraphPad Prism 6.0 (GraphPad Software, CA, USA). *P*-values <0.05 were considered to be significant.

## Results

### Identification and functional annotation of DEGs

After applying thresholds of |log_2_ FC| > 1 and *P*<0.01, we identified 1706 DEGs, including 859 up-regulated and 847 down-regulated genes, between tamoxifen-sensitive and tamoxifen-resistant breast cancer samples. A volcano plot of the DEGs is shown in Supplementary Figure S1.

According to GO enrichment analysis, up-regulated genes were significantly enriched in various biological processes (BPs) including ‘protein targeting to the ER’. The down-regulated genes were primarily enriched in ‘signal release’ and ‘positive regulation of hormone secretion’ (Supplementary Figure S2). According to KEGG analysis of the up-regulated DEGs, ‘ribosome’ and ‘oxidative phosphorylation’ were the most obviously enriched keywords (Supplementary Figure S3).

### Weighted co-expression network construction and key module identification

**Figure 1:**
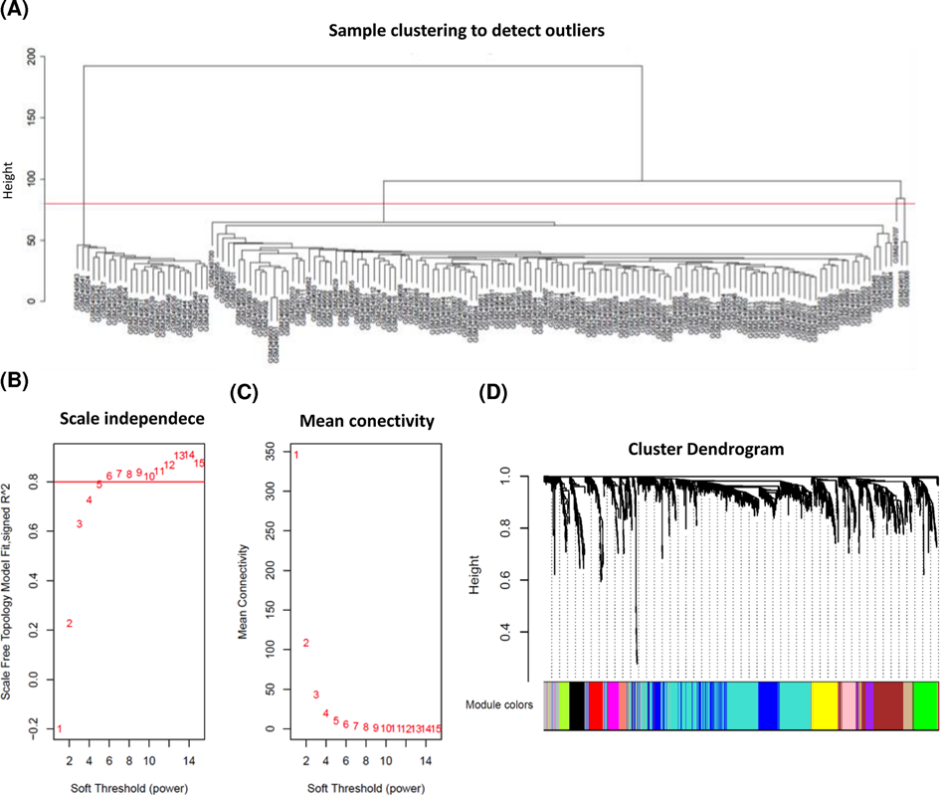
Clustering of samples and determination of soft-thresholding power in the WGCNA (**A**) Samples clustering of GSE9893 to detect outliers. A total of 28 samples were excluded. (**B**) Analysis of the scale-free fit index for soft-thresholding powers (β) from 1 to 15. (**C**) Analysis of the mean connectivity for various β values. β = 6 was chosen for subsequent analyses. (**D**) A tree map of GSE9893 gene cluster. A total of 13 co-expression modules were constructed and displayed in different colors.

**Figure 2:**
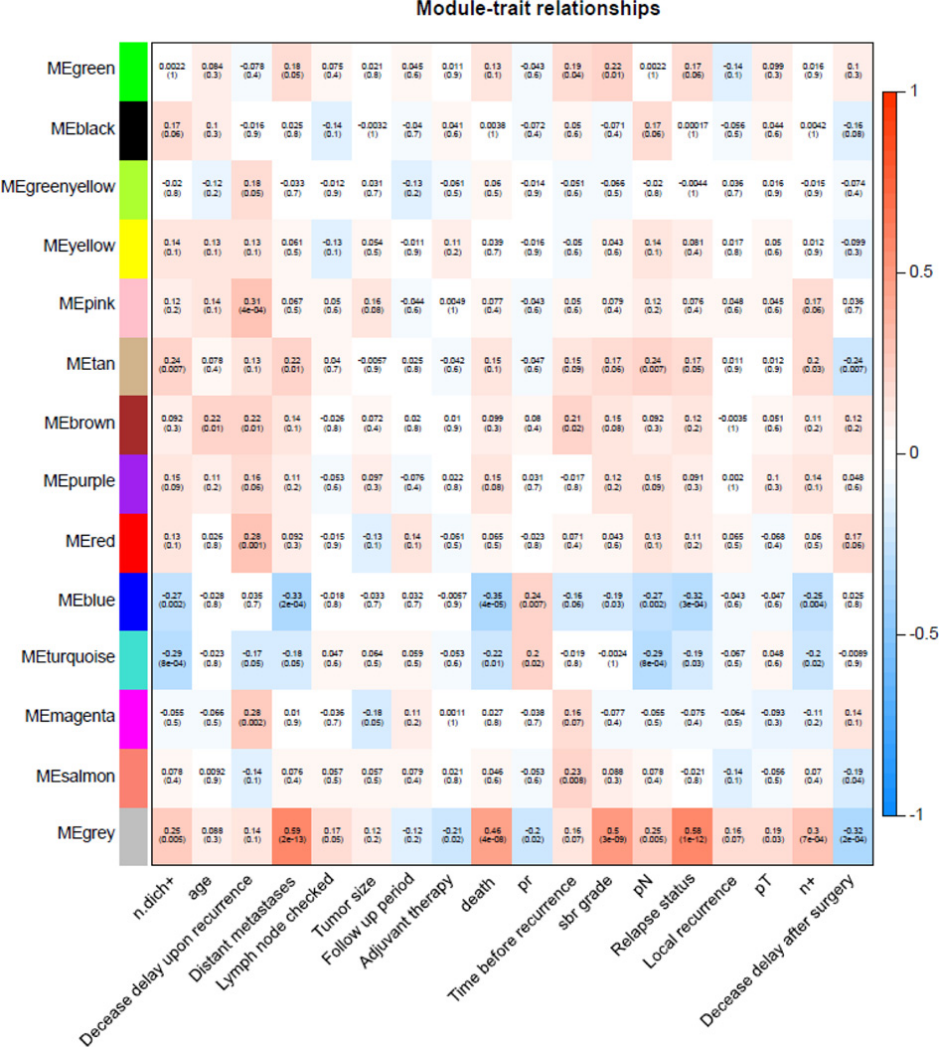
Hub modules selection Each unit contains the corresponding correlation coefficient and *P*-value. Among them, the tan and blue modules were the most relevant modules with cancer traits.

In the present study, 28 abnormal samples were excluded (Figure 1A). The value of soft-thresholding powers (β) = 6 was selected to achieve a relatively scale-free network, which was closer to the real biological network state (Figure 1B,C). We then identified 14 modules via average linkage hierarchical clustering. The DEGs in gray were not included in any module; therefore, we did not perform any functional analysis of the DEGs in gray (Figure 1D). Of these modules, the tan module showed obvious positive correlations with relapse, distant metastasis, and death. Moreover, a significant negative correlation was found between the blue module and poor prognosis (Figure 2). Hence, the tan and blue modules may play essential roles in the BPs of breast cancer tamoxifen resistance. Thus, these modules, as the most related to disease progression, were chosen for further analysis.

### Identification of hub genes in the tan and blue modules

**Figure 3:**
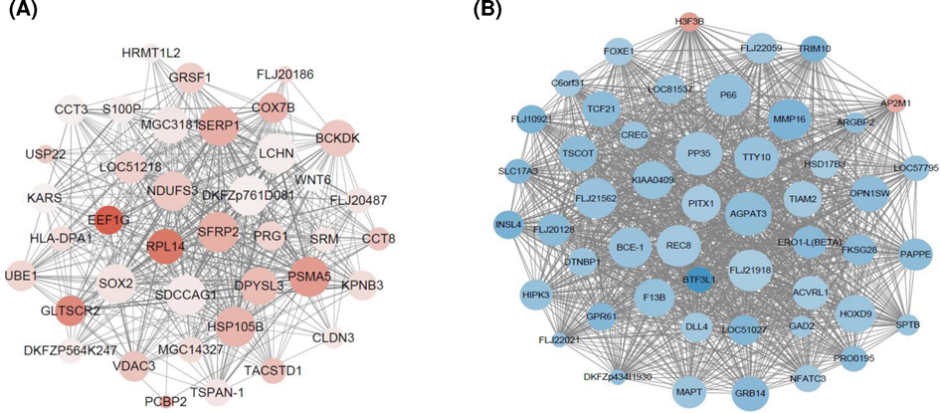
The visualization of hub genes (**A**) Genes from tan module. (**B**) Genes from blue module. The red nodes represent up-regulated genes and the blue nodes represent down-regulated genes. Node size is correlated with the degree of connectivity for the corresponding gene.

Hub genes have high connectivity within clinic-related modules, and tend to play critical roles in the molecular mechanisms of tamoxifen resistance. Therefore, we next used Cytoscape to visualize hub gene networks in the tan and blue modules. As shown in Figure 3, 38 and 50 genes with the highest intramodular connectivity in the tan and blue modules, respectively, were screened.

### Identification of prognosis-related hub genes

**Figure 4:**
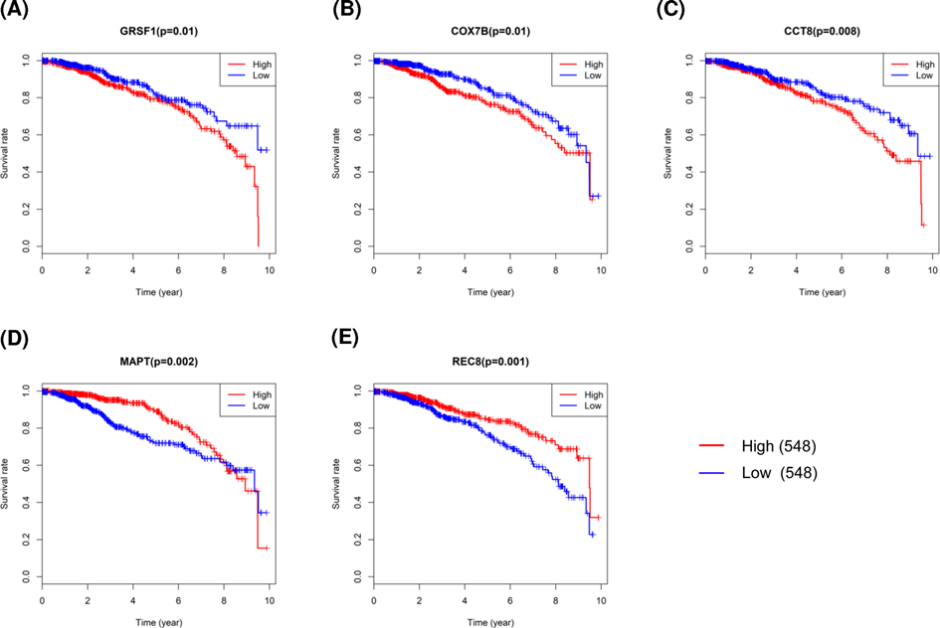
Survival analysis of prognosis-related hub genes in breast cancer patients from TCGA dataset (**A**) GRSF1. (**B**) COX7B. (**C**) CCT8. (**D**) MAPT. (**E**) REC8.

To further explore the effects of these candidate key genes on prognosis in breast cancer, we conducted survival analysis of 88 hub genes based on TCGA data. High expression of three hub genes, i.e. *GRSF1*, cytochrome *c* oxidase subunit 7B (*COX7B*), and chaperonin containing TCP1 subunit 8 (*CCT8*), in the tan module were all significantly associated with poor survival outcomes, whereas *MAPT* and REC8 meiotic recombination protein (*REC8*) in the blue module all predicted better prognosis in breast cancer when overexpressed (Figure 4). Thus, these five genes were chosen as candidates for further study.

### Validation of prognosis-related hub genes in three ER-positive breast cancer cohorts

Subsequently, we validated the prognostic relevance of the five selected hub genes (*GRSF1*, *COX7B*, *CCT8*, *MAPT*, and *REC8*) based on three independent ER-positive breast cancer cohorts (GSE9893, GSE3494, and GSE25066 datasets) involving 652 patients. Two of the five genes, i.e., *GRSF1* and *MAPT*, remained significantly associated with prognosis in patients with ER-positive breast cancer in these datasets (*P*<0.05; Table 1).

**Table 1:** Association between five candidate genes with survival in three independent ER positive breast cancer cohorts

Gene	GSE9893 cohort			GSE3494 ER^+^ cohort			GSE25066 ER^+^ cohort		
	HR	95% CI	*P*-value	HR	95% CI	*P*-value	HR	95% CI	*P*-value
*GRSF1*	1.38	1.19–1.6	<0.001**	2.01	1.02–3.97	0.044*	2.02	1.05–3.90	0.032*
*COX7B*	1.25	1.11–1.41	0.0002**	2.26	1.01–5.05	0.047*	1.83	0.97–3.45	0.056
*CCT8*	1.29	1.14–1.45	<0.001**	2.13	1.07–4.22	0.031*	1.78	0.94–3.34	0.071
*MAPT*	0.72	0.61–0.83	<0.001**	0.75	0.62–0.9	0.002**	0.34	0.18–0.67	0.001**
*REC8*	0.58	0.46–0.73	<0.001**	0.71	0.31–1.66	0.434	2.55	1.33–4.92	0.0036**

*P<0.05 and **P<0.01.

### Validation of hub genes *in vitro*

**Figure 5:**
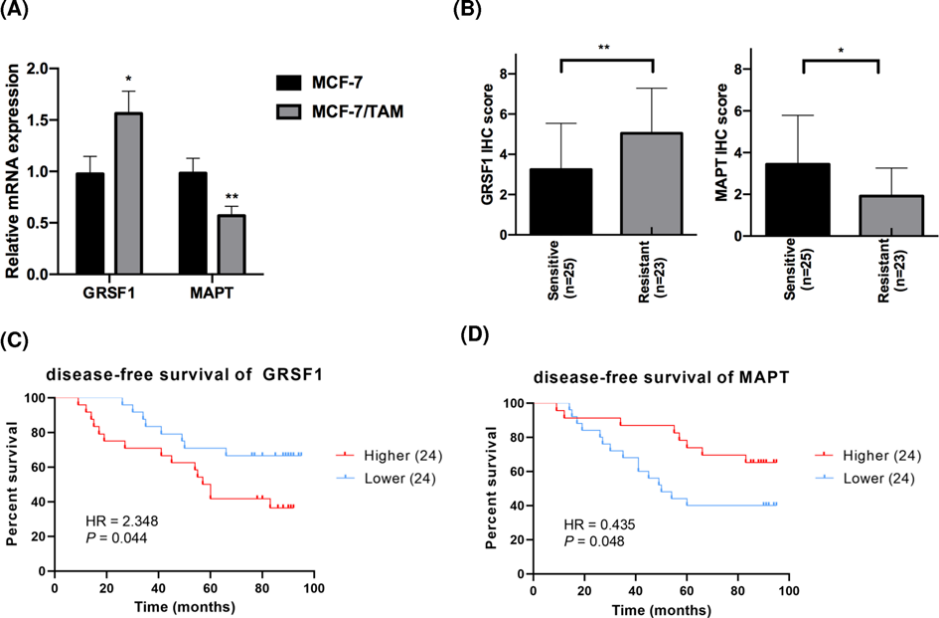
Validation of prognosis-related hub genes *in vitro* (**A**) RT-qPCR results of GRSF1 and MAPT in tamoxifen-resistant/sensitive MCF-7 subclones. (**B**) Comparison of GRSF1/MAPT expression levels detected by IHC in breast cancer tissues according to their sensitivities to tamoxifen. (**C**,**D**) Survival analysis of DFS based on GRSF1/MAPT levels using 48 tamoxifen-treated breast cancer patients. *P<0.05 and **P<0.01.

Finally, we applied RT-qPCR to further verify the expression of candidate hub genes in breast cancer cell lines. The expression level of *GRSF1* was higher in tamoxifen-resistant cells, whereas *MAPT* expression levels were significantly elevated in MCF-7 cells compared with parental MCF-7/TAM cells (Figure 5A). Similarly, the aberrant expression pattern of GRSF1 and MAPT was also verified by immunohistochemical (IHC) staining in 48 clinical tissue samples (23 tamoxifen-resistant versus 25 tamoxifen-sensitive) collected from breast cancer patients receiving tamoxifen treatment in our hospital (GRSF1: *P*=0.009; MAPT: *P*=0.017, Figure 5B). Survival analysis demonstrated that up-regulated GRSF1 expression was significantly associated with poorer disease-free survival (DFS) (HR = 2.348, 95% CI: 1.032–5.346, *P*=0.044, Figure 5C), and MAPT has the capacity to predict favorable DFS in these patients (HR = 0.435, 95% CI: 0.191–0.988, *P*=0.048, Figure 5D). The data of these independent experiments therefore verified the hypotheses generated using bioinformatics analysis, indicating that GRSF1 and MAPT might play a vital role in tamoxifen resistance in breast cancer.

### GSEA

**Figure 6:**
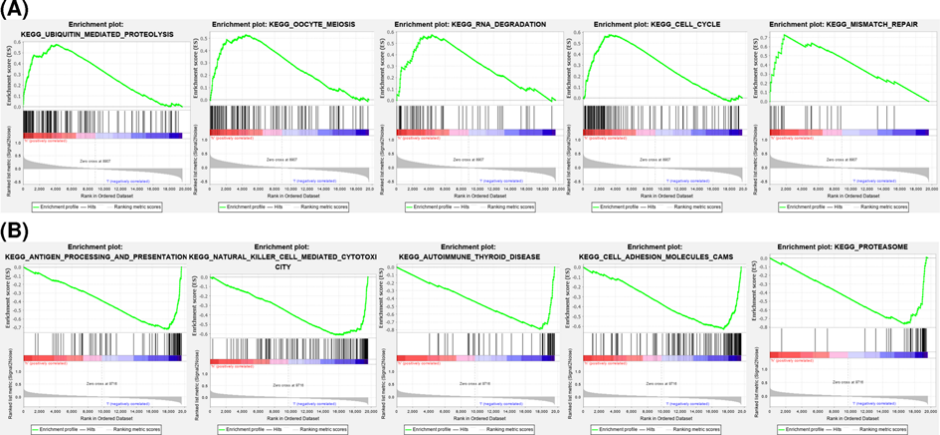
GSEA (**A**) The top five enriched entries of GRSF1. (**B**) The top five enriched entries of MAPT.

To better understand the underlying function of these hub genes, GSEA was carried out and mapped on to KEGG pathways. As illustrated in Figure 6, *GRSF1* was mostly involved in ‘ubiquitin-mediated proteolysis’, ‘oocyte meiosis’, ‘RNA degradation’, ‘cell cycle’, and ‘mismatch repair’. *MAPT* was related to ‘antigen processing and presentation’, ‘natural killer cell-mediated cytotoxicity’, ‘autoimmune thyroid disease’, ‘cell adhesion molecules’, and the ‘proteasome’.

### Association of hub gene expression and immune infiltration level

**Figure 7:**
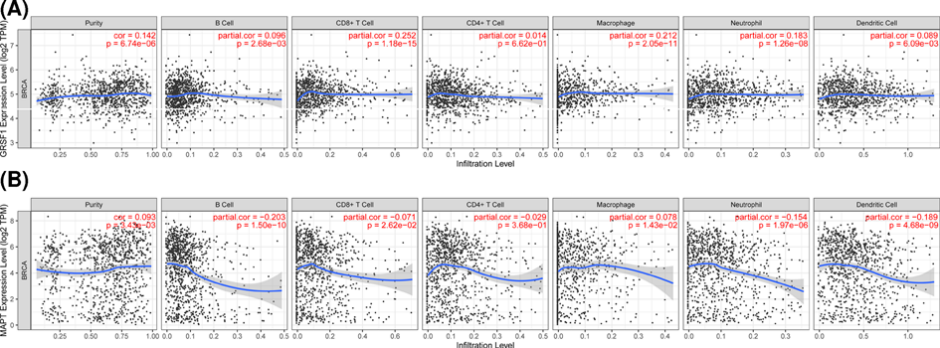
The correlation between hub genes and immune cell infiltration levels in breast cancer through TIMER (**A**) Association between GRSF1 and immune cells. (**B**) Association between MAPT and immune cells.

The distribution of tumor-infiltrating cells is highly relevant to tumor progression. Therefore, we evaluated the association of hub genes with immune infiltration level using the TIMER platform. The level of GRSF1 expression was positively correlated with the abundance of infiltrating immune cells. While MAPT expression displayed a significant negative correlation with infiltration degree by B cells, CD4 T cells, CD8 T cells, neutrophils, and dendritic cells (Figure 7). These findings suggest that GRSF1 and MAPT may be involved in immune infiltration in patients with breast cancer.

## Discussion

ER-positive breast cancer exhibits a favorable prognosis owing to the efficacy of anti-estrogen drugs, such as tamoxifen [1]. However, one-third of these patients eventually develop tamoxifen resistance, resulting in cancer progression and death [5,15]. Tamoxifen resistance occurs via a complicated series of events, taking place over multiple genes and various signaling pathways. An in-depth elucidation of the biological mechanisms of tamoxifen insensitivity is beneficial to identify novel prognostic biomarkers, and explore effective therapeutic targets towards overcoming tamoxifen resistance. Due to the establishment of large cancer databases, such as the TCGA and GEO databases, researchers have the capacity to investigate large-scale gene expression profiles [16]. In the present study, we screened for hub genes involved in the development of tamoxifen insensitivity that could be used as potential biomarkers to predict tamoxifen response and prognosis in ER-positive breast cancer patients.

In the present study, we first identified 1706 DEGs associated with tamoxifen resistance, including 859 up-regulated and 847 down-regulated genes. These DEGs were primarily enriched in functions such as protein targeting the ER and pathways such as oxidative phosphorylation. As a hormonal transcription factor, ERs regulate target genes to manipulate cell cycle progression and the endocrine response. The activity of ERs is also regulated by multiple proteins, including the transcription factors Ap-1 and FOXA1, which exert different biological functions in response to endocrine treatment [17–19]. Meanwhile, many studies have shown that oxidative phosphorylation is closely correlated with carcinogenesis. Echeverria et al*.* reported that an oxidative phosphorylation inhibitor delayed residual tumor regrowth for neoadjuvant chemotherapy-resistant patients with breast cancer [20]. Sansone et al*.* demonstrated that the activation of oxidative phosphorylation promoted the development of hormone therapy-resistant disease [21]. Overall, these studies imply that the DEGs identified in the present study might be closely connected with tumor progression and endocrine efficiency.

Subsequently, we utilized WGCNA to filter highly reliable and biologically significant modules and hub genes that are responsible for tamoxifen resistance from the list of DEGs [22,23]. The WGCNA clustered genes into 13 modules, of which the tan and blue modules were positively and negatively related to clinical traits, respectively. From this analysis, hub genes in these two modules were selected. Subsequent survival analysis showed that high expression of *GRSF1* predicted poor prognosis, whereas *MAPT* was associated with favorable survival outcomes in TCGA breast cancer patients. Notably, we further verified the prognostic value of candidate hub genes in three independent ER-positive breast cancer cohorts. Compared with previous studies [15,25], we found modules and genes that were relevant to malignant phenotypes and favorable clinical features. More importantly, we validated the hypotheses generated by the available databases using tamoxifen-sensitive and tamoxifen-resistant cell lines as well as clinical tissue specimens. We thus identified *GRSF1* and *MAPT* as the most promising candidate genes related to tamoxifen resistance. Subsequently, we further explored the potential roles of hub genes in ER-positive breast cancer using GSEA software. The results showed that several pathways such as ‘RNA degradation’ and ‘cell cycle’ were dysregulated when *GRSF1* was aberrantly expressed. The potential mechanism of *MAPT* is strongly linked to immune-related signaling pathways such as ‘antigen processing and presentation’, ‘natural killer cell-mediated cytotoxicity’, and ‘cytokine–cytokine receptor interaction’. TIMER analysis also indicated a correlation between *MAPT* expression and immune infiltration, suggesting that *MAPT* might have a function in tumor immunity.

GRSF1 was initially identified as an RNA-binding protein with high affinity for G-rich sequences. GRSF1 plays critical roles in maintaining mitochondrial function, including mitochondrial translation, mitochondrial ribosome biosynthesis, and mitochondrial noncoding RNA binding [24,25]. At present, only a small number of studies have focused on the role of GRSF1 in cancer. Sun et al*.* and Yang et al*.* demonstrated that GRSF1 regulates miRNAs to facilitate oncogenic behaviors, including autophagy and metastasis, in cervical cancer [26,27]. Wang et al*.* revealed that GRSF1 can accelerate tumorigeneis and metastasis via PI3K/AKT pathway in gastric cancer [28]. Taken together with the results of the present study, these findings imply that GRSF1 might function as a potential oncogene.

*MAPT* is a gene encoding τ protein, which is implicated in the pathogenesis of several neurodegenerative disorders such as Alzheimer’s disease, Parkinson’s disease, and progressive supranuclear palsy [29,30]. Recent studies have suggested that elevated expression of *MAPT* predicts better survival outcomes in pediatric neuroblastoma, breast cancer, renal clear cell cancer, and low-grade glioma, which is consistent with the results of the present study [31–34]. Wang et al. reported that MAPT-hypermethylated tumors are closely associated with poor prognosis in patients with colorectal cancer [35]. Interestingly, MAPT is reported to play an essential role in mediating paclitaxel or taxane resistance in various cancers. Rouzier et al*.* first identified MAPT as a predictor of the response to paclitaxel in breast cancer [36]. MAPT can also determine paclitaxel chemosensitivity by interacting with several miRNAs in gastric cancer and non-small cell lung cancer [37]. Moreover, clinical and *in vitro* studies have demonstrated that the expression level of MAPT is positively associated with ER expression, and is influenced by ER signaling [38,39]. Taken together, these studies indicate that MAPT clearly plays a complex and possibly cancer-specific role in different cancers, which warrants more in-depth, well-designed investigations.

However, the present study had some limitations. First, tamoxifen resistance is controlled by a complicated regulatory network comprising mRNAs, miRNAs, and long noncoding RNAs; however, since we were restricted by the available datasets, only protein-coding genes/mRNAs were included in the present analysis. The precise roles of MAPT and GRSF1 may only become clear in the context of miRNAs and long noncoding RNAs. Second, despite the validation of key genes in cancer cell lines and tissue samples, our model has not been verified in a sufficiently large clinical cohort, or prospective individual cohorts. Third, we predicted the possible functions of specific genes using the available network information, but the underlying mechanisms of gene networks involved in tamoxifen response warrant further study.

## Conclusion

The present study identified gene networks and potential prognostic biomarkers using a systems biology-based WGCNA approach in patients with primary breast cancer treated with tamoxifen. Through a series of bioinformatics analyses and preliminary biological experiments, we identified and verified two novel biomarkers that may be related to the tamoxifen response in ER-positive breast cancer: *GRSF1*, a prognostic marker for cancer progression, and *MAPT*, to predict favorable survival outcomes. GSEA suggested that *GRSF1* might be involved in RNA degradation and cell cycle pathways, while *MAPT* was closely linked to immune-related signaling pathways. However, further studies are needed to elucidate the exact molecular mechanisms and characterize the key genes functionally affecting tamoxifen sensitivity in patients with breast cancer.

## Supplementary Material

Supplementary Figures S1-S3

## Data Availability

Publicly available datasets were analyzed in the present study, these can be obtained from GEO (GSE9893, GSE3494, GSE25066) and TCGA.

## Abbreviations

BP: biological process
*CCT8*: chaperonin containing TCP1 subunit 8
*COX7B*: cytochrome *c* oxidase subunit 7B
DEG: differentially expressed gene
DFS: disease-free survival
ER: estrogen receptor
GO: Gene Ontology
*GRSF1*: G-rich RNA sequence binding factor 1
GSEA: gene set enrichment analysis
KEGG: Kyoto Encyclopedia of Genes and Genomes
*MAPT*: microtubule-associated protein τ
*REC8*: REC8 meiotic recombination protein
RT-qPCR: reverse transcription quantitative polymerase chain reaction
TCGA: The Cancer Genome Atlas
TIMER: tumor immune estimation resource
TOM: topological overlap matrix
WGCNA: weighted gene co-expression network analysis

## References

[1] Perou C.M., Sorlie T., Eisen M.B., van de Rijn M., Jeffrey S.S., Rees C.A. et al. (2000) Molecular portraits of human breast tumours. Nature 406, 747–752 10.1038/35021093 10963602

[2] Early Breast Cancer Trialists' Collaborative, G (1988) Effects of adjuvant tamoxifen and of cytotoxic therapy on mortality in early breast cancer. An overview of 61 randomized trials among 28,896 women. N. Engl. J. Med. 319, 1681–1692 10.1056/NEJM198812293192601 3205265

[3] Early Breast Cancer Trialists’ Collaborative Group (2005) Effects of chemotherapy and hormonal therapy for early breast cancer on recurrence and 15-year survival: an overview of the randomised trials. Lancet 365, 1687–1717 10.1016/S0140-6736(05)66544-0 15894097

[4] Fan W., Chang J. and Fu P. (2015) Endocrine therapy resistance in breast cancer: current status, possible mechanisms and overcoming strategies. Future Med. Chem. 7, 1511–1519 10.4155/fmc.15.93 26306654 PMC5558537

[5] Ali S. and Coombes R.C. (2002) Endocrine-responsive breast cancer and strategies for combating resistance. Nat. Rev. Cancer 2, 101–112 10.1038/nrc721 12635173

[6] Horvath S., Zhang B., Carlson M., Lu K.V., Zhu S., Felciano R.M. et al. (2006) Analysis of oncogenic signaling networks in glioblastoma identifies ASPM as a molecular target. Proc. Natl. Acad. Sci. U.S.A. 103, 17402–17407 10.1073/pnas.0608396103 17090670 PMC1635024

[7] Langfelder P. and Horvath S. (2008) WGCNA: an R package for weighted correlation network analysis. BMC Bioinformatics 9, 559 10.1186/1471-2105-9-559 19114008 PMC2631488

[8] Jin Y. and Qin X. (2020) Co-expression network-based identification of biomarkers correlated with the lymph node metastasis of patients with head and neck squamous cell carcinoma. Biosci. Rep. 40, BSR20194067 10.1042/BSR20194067 32076707 PMC7033310

[9] Tang J., Kong D., Cui Q., Wang K., Zhang D., Gong Y. et al. (2018) Prognostic genes of breast cancer identified by gene co-expression network analysis. Front. Oncol. 8, 374 10.3389/fonc.2018.00374 30254986 PMC6141856

[10] Jiang C., Wu S., Jiang L., Gao Z.C., Li X.R., Duan Y.Y. et al. (2019) Network-based approach to identify biomarkers predicting response and prognosis for HER2-negative breast cancer treatment with taxane-anthracycline neoadjuvant chemotherapy. PeerJ 7, e7515 10.7717/peerj.7515 31534839 PMC6730536

[11] Barrett T., Troup D.B., Wilhite S.E., Ledoux P., Rudnev D., Evangelista C. et al. (2007) NCBI GEO: mining tens of millions of expression profiles–database and tools update. Nucleic Acids Res. 35, D760–D765 10.1093/nar/gkl887 17099226 PMC1669752

[12] Shannon P., Markiel A., Ozier O., Baliga N.S., Wang J.T., Ramage D. et al. (2003) Cytoscape: a software environment for integrated models of biomolecular interaction networks. Genome Res. 13, 2498–2504 10.1101/gr.1239303 14597658 PMC403769

[13] Subramanian A., Tamayo P., Mootha V.K., Mukherjee S., Ebert B.L., Gillette M.A. et al. (2005) Gene set enrichment analysis: A knowledge-based approach for interpreting genome-wide expression profiles. Proc. Natl. Acad. Sci. U.S.A. 102, 15545–15550 10.1073/pnas.0506580102 16199517 PMC1239896

[14] Li T., Fan J., Wang B., Traugh N., Chen Q., Liu J.S. et al. (2017) TIMER: a web server for comprehensive analysis of tumor-infiltrating immune cells. Cancer Res. 77, E108–E110 10.1158/0008-5472.CAN-17-0307 29092952 PMC6042652

[15] Orcurto A., Odermatt R., Stravodimou A. and Wolfer A. (2014) Endocrine therapy resistance in metastatic breast cancer: mechanisms and clinical implications. Rev. Med. 10, 1102–1106 24941677

[16] Weinstein J.N., Collisson E.A., Mills G.B., Shaw K.R.M., Ozenberger B.A., Ellrott K. et al. (2013) The Cancer Genome Atlas Pan-Cancer analysis project. Nat. Genet. 45, 1113–1120 10.1038/ng.2764 24071849 PMC3919969

[17] Nilsson S., Makela S., Treuter E., Tujague M., Thomsen J., Andersson G. et al. (2001) Mechanisms of estrogen action. Physiol. Rev. 81, 1535–1565 10.1152/physrev.2001.81.4.1535 11581496

[18] Hurtado A., Holmes K.A., Ross-Innes C.S., Schmidt D. and Carroll J.S. (2011) FOXA1 is a key determinant of estrogen receptor function and endocrine response. Nat. Genet. 43, 27–33 10.1038/ng.730 21151129 PMC3024537

[19] Wu Y.M., Zhang Z., Cenciarini M.E., Proietti C.J., Amasino M., Hong T. et al. (2018) Tamoxifen resistance in breast cancer is regulated by the EZH2-ER alpha-GREB1 transcriptional axis. Cancer Res. 78, 671–684 10.1158/0008-5472.CAN-17-1327 29212856 PMC5967248

[20] Echeverria G.V., Ge Z., Seth S., Zhang X., Jeter-Jones S., Zhou X. et al. (2019) Resistance to neoadjuvant chemotherapy in triple-negative breast cancer mediated by a reversible drug-tolerant state. Sci. Transl. Med. 11, eaav0936 10.1126/scitranslmed.aav0936 30996079 PMC6541393

[21] Sansone P., Savini C., Kurelac I., Chang Q., Amato L.B., Strillacci A. et al. (2017) Packaging and transfer of mitochondrial DNA via exosomes regulate escape from dormancy in hormonal therapy-resistant breast cancer. Proc. Natl. Acad. Sci. U.S.A. 114, E9066–E9075 10.1073/pnas.1704862114 29073103 PMC5664494

[22] Yang Y., Qi S., Shi C., Han X., Yu J., Zhang L. et al. (2020) Identification of metastasis and prognosis-associated genes for serous ovarian cancer. Biosci. Rep. 40, BSR20194324 10.1042/BSR20194324 32510146 PMC7317593

[23] Tang J., Lu M., Cui Q., Zhang D., Kong D., Liao X. et al. (2019) Overexpression of ASPM, CDC20, and TTK confer a poorer prognosis in breast cancer identified by gene co-expression network analysis. Front. Oncol. 9, 310 10.3389/fonc.2019.00310 31106147 PMC6492458

[24] Antonicka H., Sasarman F., Nishimura T., Paupe V. and Shoubridge E.A. (2013) The mitochondrial RNA-binding protein GRSF1 localizes to RNA granules and is required for posttranscriptional mitochondrial gene expression. Cell Metab. 17, 386–398 10.1016/j.cmet.2013.02.006 23473033

[25] Jourdain A.A., Koppen M., Wydro M., Rodley C.D., Lightowlers R.N., Chrzanowska-Lightowlers Z.M. et al. (2013) GRSF1 regulates RNA processing in mitochondrial RNA granules. Cell Metab. 17, 399–410 10.1016/j.cmet.2013.02.005 23473034 PMC3593211

[26] Sun Q., Yang Z., Li P., Wang X., Sun L., Wang S. et al. (2019) A novel miRNA identified in GRSF1 complex drives the metastasis via the PIK3R3/AKT/NF-kappaB and TIMP3/MMP9 pathways in cervical cancer cells. Cell Death Dis. 10, 636 10.1038/s41419-019-1841-5 31474757 PMC6717739

[27] Yang Z., Sun Q., Guo J., Wang S., Song G., Liu W. et al. (2019) GRSF1-mediated MIR-G-1 promotes malignant behavior and nuclear autophagy by directly upregulating TMED5 and LMNB1 in cervical cancer cells. Autophagy 15, 668–685 10.1080/15548627.2018.1539590 30394198 PMC6526811

[28] Wang B., Wang L., Lu Y., Liang W., Gao Y., Xi H. et al. (2021) GRSF1 promotes tumorigenesis and EMT-mediated metastasis through PI3K/AKT pathway in gastric cancer. Biochem. Biophys. Res. Commun. 555, 61–66 10.1016/j.bbrc.2021.03.121 33813277

[29] Bloom G.S. (2014) Amyloid-beta and tau the trigger and bullet in Alzheimer disease pathogenesis. JAMA Neurol. 71, 505–508 10.1001/jamaneurol.2013.5847 24493463 PMC12908160

[30] Caillet-Boudin M.-L., Buee L., Sergeant N. and Lefebvre B. (2015) Regulation of human MAPT gene expression. Mol. Neurodegen. 10, 28 10.1186/s13024-015-0025-8 PMC4499907 26170022

[31] Bonneau C., Gurard-Levin Z.A., Andre F., Pusztai L. and Rouzier R. (2015) Predictive and prognostic value of the tau protein in breast cancer. Anticancer Res. 35, 5179–5184 26408675

[32] Zaman S., Chobrutskiy B.I. and Blanck G. (2018) MAPT (Tau) expression is a biomarker for an increased rate of survival in pediatric neuroblastoma. Cell Cycle 17, 2474–2483 10.1080/15384101.2018.1542898 30394813 PMC6342068

[33] Han X., Sekino Y., Babasaki T., Goto K., Inoue S., Hayash T. et al. (2020) Microtubule-associated protein tau (MAPT) is a promising independent prognostic marker and tumor suppressive protein in clear cell renal cell carcinoma. Urol. Oncol. 38, 605.e9–605.e17 10.1016/j.urolonc.2020.02.010 32139291

[34] Zaman S., Chobrutskiy B.I., Sikaria D. and Blanck G. (2019) MAPT (Tau) expression is a biomarker for an increased rate of survival for low-grade glioma. Oncol. Rep. 41, 1359–1366 30535461 10.3892/or.2018.6896

[35] Wang C., Liu Y., Guo W., Zhu X., Ahuja N. and Fu T. (2019) MAPT promoter CpG island hypermethylation is associated with poor prognosis in patients with stage II colorectal cancer. Cancer Manag. Res. 11, 7337–7343 10.2147/CMAR.S206731 31496795 PMC6689138

[36] Rouzier R., Rajan R., Wagner P., Hess K.R., Gold D.L., Stec J. et al. (2005) Microtubule-associated protein tau: A marker of paclitaxel sensitivity in breast cancer. Proc. Natl. Acad. Sci. U.S.A. 102, 8315–8320 10.1073/pnas.0408974102 15914550 PMC1149405

[37] Cai Y., Jia R., Xiong H., Ren Q., Zuo W., Lin T. et al. (2019) Integrative gene expression profiling reveals that dysregulated triple microRNAs confer paclitaxel resistance in non-small cell lung cancer via co-targeting MAPT. Cancer Manag. Res. 11, 7391–7404 10.2147/CMAR.S215427 31496800 PMC6689126

[38] Pentheroudakis G., Kalogeras K.T., Wirtz R.M., Grimani I., Zografos G., Gogas H. et al. (2009) Gene expression of estrogen receptor, progesterone receptor and microtubule-associated protein Tau in high-risk early breast cancer: a quest for molecular predictors of treatment benefit in the context of a Hellenic Cooperative Oncology Group trial. Breast Cancer Res. Treat. 116, 131–143 10.1007/s10549-008-0144-9 18668363

[39] Ikeda H., Taira N., Hara F., Fujita T., Yamamoto H., Soh J. et al. (2010) The estrogen receptor influences microtubule-associated protein tau (MAPT) expression and the selective estrogen receptor inhibitor fulvestrant downregulates MAPT and increases the sensitivity to taxane in breast cancer cells. Breast Cancer Res. 12, R43 10.1186/bcr2598 20579400 PMC2917038

