# Supplementary material for: Network-based approach to identify prognosis-related genes in tamoxifen-treated patients with estrogen receptor-positive breast cancer
Source: Biosci Rep. 2021 Sep 3;41(9):BSR20203020. doi: 10.1042/BSR20203020 (PMC8485391; doi:10.1042/BSR20203020)
Supplement: Supplementary Figures S1-S3 [file BSR-2020-3020_supp.pdf]

Supplementary data

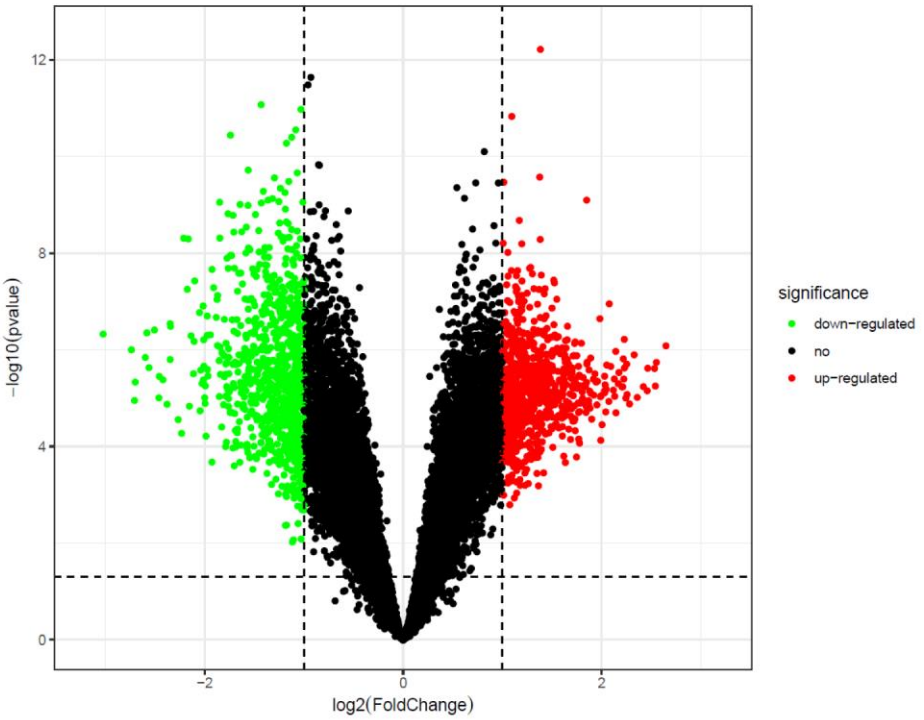

Figure S1. Identification of DEGs in GSE9893. Volcano plot of the aberrantly expressed genes between tamoxifen sensitive and resistant breast cancer groups.

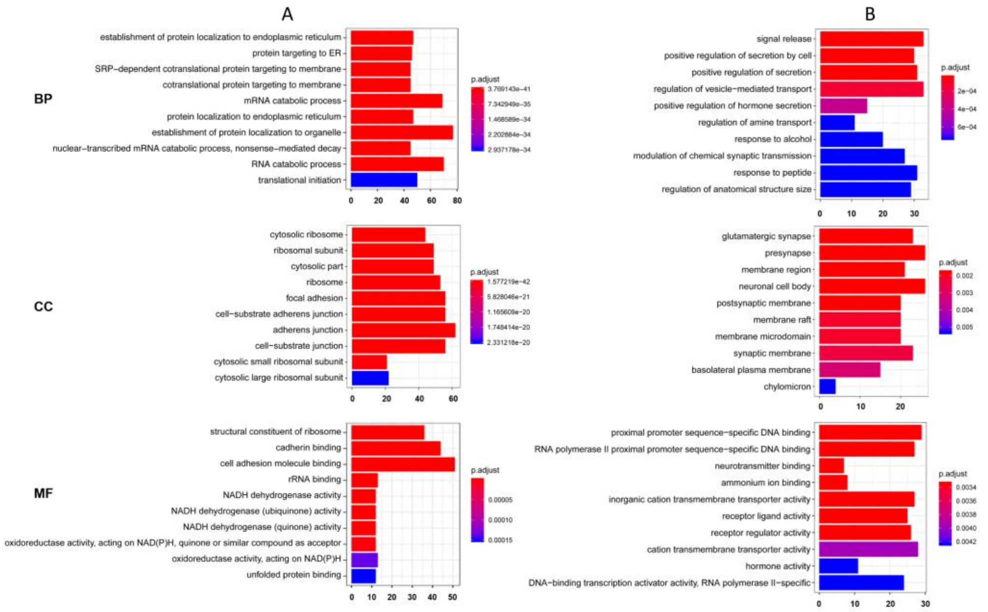

Figure S2. Go annotations of DEGs. (A) Upregulated DEGs. (B) Downregulated DEGs. BP: biological process, CC: cellular component, MF: molecular function.

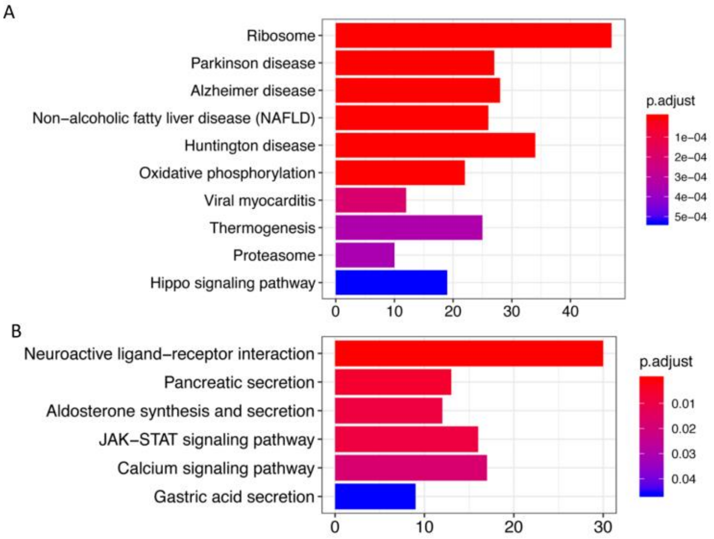

Figure S3. KEGG enrichment analysis of DEGs. (A) Upregulated DEGs. (B) Downregulated DEGs.
